# Dynamic pillar–perfusion platform for screening enzyme‐induced self‐assembling peptide therapeutics in 3D breast cancer spheroids

**DOI:** 10.1002/btm2.70135

**Published:** 2026-04-09

**Authors:** Andrea Escobar Martinez, Pranav Joshi, Emily Carney, Faye Fouladgar, Robert Powell, Manav Goud Vanga, Victoria Gnenema, Sarah Hripko, Moo‐Yeal Lee, Neda Habibi

**Affiliations:** ^1^ Biomedical Engineering Department University of North Texas Denton Texas USA; ^2^ Bioprinting Laboratories Inc. Dallas Texas USA

**Keywords:** breast cancer, peptide, pillar‐plate, self‐assembling, spheroid

## Abstract

Enzyme‐induced self‐assembling peptides (EISAPs) are a promising class of enzyme‐activated anticancer therapeutics, yet their translational screening is limited by the lack of 3D tumor models that effectively capture drug penetration, self‐assembly dynamics, and treatment response. To address this need, we developed a pillar–perfusion 3D breast cancer spheroid platform to screen a six‐peptide panel—P1 (Fmoc‐FF‐pTyr), P2 (Fmoc‐FF‐pThr), P3 (RGD‐FF‐pTyr), P4 (NBD‐FF‐pTyr), P5 (Nap‐FF‐pTyr), and P6 (Nap‐FF‐pThr)—under static and dynamic flow. Hydrogel optimization identified a 2% gelatin/1% alginate matrix enabling *>*90% spheroid transfer efficiency and stable non‐invasive morphology, while Matrigel‐based embedding generated invasive spheroids mimicking metastatic behavior. Across the peptide panel, P1 and P5 produced the strongest cytotoxic effects, with dynamic perfusion further enhancing activity (P1 viability *∼*55% at 100 μM). Co‐treatment with P1 + 5 μM Doxorubicin resulted in enhanced viability loss and complete inhibition of invasion. Fluorescence imaging of NBD‐FF‐pTyr confirmed progressive intratumoral penetration and core accumulation over 5 days. RT‐qPCR analysis demonstrated peptide‐ and subtype‐specific transcriptional effects, with P1 in MCF‐7 spheroids significantly downregulating *BCL2*, *BRCA2*, and *TP53*, while P5 in MDA‐MB‐231 spheroids produced the strongest repression of survival and DNA‐repair pathways. These findings establish a dynamic, high‐throughput 3D platform for comparative EISAP screening and demonstrate the therapeutic potential of enzyme‐responsive peptides in complex tumor microenvironments.

## Graphical abstract

Enzyme‐instructed self‐assembling peptides suppress breast cancer spheroid invasion and outgrowth in 3D microenvironments through intracellular activation and nanostructure formation. Using pillar perfusion culture, enhanced cytotoxicity and therapeutic response were observed under dynamic conditions, highlighting an effective strategy to overcome resistance in tumor spheroid models.

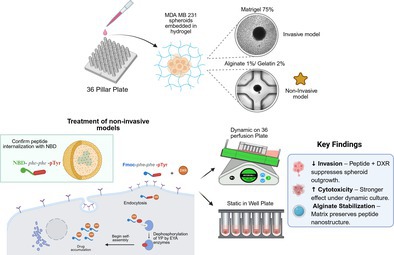

**Translational Impact Statement**

This work developed a pillar–perfusion system that mimics the 3D tumor environment and provides dynamic conditions similar to fluid flow in tumors. An optimized hydrogel allowed transfer and support of spheroids on the pillars. The platform enabled screening of peptide therapeutics in both stable and invasive breast cancer spheroids. The results generated significant data on the penetration of peptides, the inhibition of metastasis, and the impact on DNA repair pathways. These results help guide the development of effective models to evaluate short anticancer peptides for breast cancer treatment.

## 1 INTRODUCTION

Although molecular profiling and targeted therapies have advanced the treatment for breast cancer, the survival rate remains an issue for aggressive subtypes including HER2‐driven tumors and triple‐negative breast cancer (TNBC).^1^ To address drug resistance in breast cancer, novel therapeutic strategies such as enzyme‐induced self‐assembling peptides (EISAP) have gained significant attention for their ability to exploit tumor‐associated enzymatic activity and assemble in situ at the disease site.^2^, ^3^ These peptides circulate in an inactive state and self‐assemble only upon cleavage or dephosphorylation by overexpressed enzymes like alkaline phosphatases (ALP) or tyrosine phosphatase in the tumor microenvironment (TME).^2^, ^4^ Once activated, these nanostructures enhance drug accumulation, cellular uptake, and sustained release, thereby improving efficacy and helping to overcome multidrug resistance.^5^ In our earlier work, for the first time, we investigated short peptides, which undergo activation through the tyrosine phosphatase activity of the Eyes Absent (EYA) enzyme in breast cancer cells.^6^, ^7^ The enzymatic activation of EISAPs by EYA tyrosine phosphatase has been validated in our prior work, including direct measurement of phosphate release following dephosphorylation by EYA2 in vitro, SEM visualization of EYA–peptide‐induced nanofibers.^6^, ^7^ Once internalized, these peptides were distributed throughout both the cytoplasm and nucleus, remaining detectable for up to 5 days, with a subset also accumulating in the mitochondria. Notably, they do not target normal epithelial cells and instead are able to trigger DNA damage response (DDR) in breast cancer cells.^6^, ^7^ These therapeutics act through the self‐assembly of precursor molecules in response to pericellular and intracellular enzymes.

The study of peptides–tissue interactions within the TME has faced limitations due to the use of animal models and traditional monolayer cultures. Two‐dimensional (2D) models have provided valuable insights into tumorigenesis and signaling; however, they fail to capture critical features such as extracellular matrix (ECM) deposition, hypoxic gradients, cell–cell interactions, and gene expression heterogeneity, ultimately yielding misleading assessments of drug efficacy.^8^ Hence, effective clinical translation and screening, as well as feasible high‐throughput three‐dimensional (3D) cell models, are needed to better replicate the native tissue environment and blood flow.^9^ Tumor spheroids closely resemble the native TME, offering improved modeling of nutrient diffusion and drug penetration.^9^ Spheroids can be generated through several techniques, including liquid overlay systems, hanging‐drop cultures, and micropatterned substrates.^10^ However, these approaches often suffer from variability in spheroid size, poor control over microenvironmental conditions, and restricted transport of nutrients, oxygen, and therapeutics.^8^, ^9^ These diffusional barriers can result in hypoxia or necrotic cores and hinder accurate evaluation of drug penetration and efficacy.^11^ Recently, pillar–perfusion platforms have emerged as a dynamic and physiologically relevant alternative. These systems enable continuous medium exchange and spatially controlled spheroid positioning, improving nutrient and oxygen delivery while preserving 3D architecture.^10^ The incorporation of continuous flow and modular design allows real‐time monitoring, multiplexed assays, and accurate mimicry of the TME, providing a suitable model for high‐throughput testing and mechanistic studies under physiologically relevant conditions.^11^

In this work, breast cancer spheroids were modeled and studied employing MDA‐MB‐231 cells and MCF‐7 cells. Spheroids formed in ultra‐low attachment (ULA) plates were transferred to a pillar plate system using various hydrogel embedding. The pillar plate supports both static and dynamic cultures. Both invasive and non‐invasive spheroid models were developed and treated with EISAPs. The effects of the EISAP, P1‐P6 with Fmoc and Nap protecting groups were evaluated under static and dynamic flow. The results highlight that across enzyme‐induced self‐assembling peptide (EISAP) designs, structural variations can strongly influence cytotoxic potential, tumor penetration, and responsiveness to dynamic culture conditions. Combination strategies with standard chemotherapeutics such as doxorubicin may further modulate these effects, highlighting the need for platforms that can systematically compare peptide assembly performance under controlled static and perfused environments. In addition, fluorescence‐tagged constructs such as NBD‐FF‐pTyr offer opportunities to track intratumoral distribution and assess how peptide assembly progresses within 3D architectures. The pillar–perfusion platform used in this study provides the necessary control and physiological relevance to evaluate EISAP behavior, delivery, and therapeutic potential within complex 3D tumor microenvironments.

## 2 EXPERIMENTAL

### 2.1 Materials

MDA‐MB‐231 cells (HTB‐26) were acquired from the American Type Culture Collection (ATCC) and cultured in Dulbecco's Modified Eagle Medium (DMEM; Gibco, Cat. No. 11‐965‐092). MCF‐7 cells were purchased from ATCC (HTB‐22) and grown in Advanced RPMI 1640 (Gibco, Cat. No. 12633012) supplemented with 9% fetal bovine serum (FBS; Gibco, Cat. No. 26140079) and 1% penicillin–streptomycin (Gibco, Cat. No. 15140122) according to the manufacturer's instructions.^12^ Ultra‐low attachment (ULA) 96‐well (S‐Bio®, Cat. No. MS‐9096VZ) and 384‐well plates (S‐Bio®, Cat. No. MS‐9384UZ), along with 384DeepWellPlate, 36PillarPlate, 36PerfusionPlate, and loading plates (Bioprint‐ing Laboratories Inc., Dallas, TX, USA),^13^ were used for spheroid formation and transfer. Hydrogels included Matrigel® Basement Membrane Matrix (Corning, Cat. No. 354230), alginic acid sodium salt (Sigma–Aldrich, Cat. No. A1112), bovine gelatin (Sigma–Aldrich, Cat. No. G9391), and Fmoc‐Phe‐Phe‐OH (Bachem, Prod. No. 4015688), with calcium chloride dihydrate (Sigma–Aldrich, Cat. No. C7902) used for alginate crosslinking. Cus‐tom synthesis of the self‐assembling peptides was performed by GenScript (Piscataway, NJ, USA). Doxorubicin hydrochloride was obtained from Thermo Fisher Scientific (Cat. No. J64000.MA). Viability and staining reagents included Cell Counting Kit‐8 (CCK‐8; Dojindo, Cat. No. CK04), LIVE/DEAD™ Viability/Cytotoxicity Kit (Thermo Fisher Scientific, Cat. No. L3224), CellEvent™ Caspase‐3/7 Green Detection Reagent (Thermo Fisher Scientific, Cat. No. C10723), tetramethylrhodamine methyl ester perchlorate (TMRM; Thermo Fisher Scientific, Cat. No. T668), and Hoechst 33342 (Thermo Fisher Scientific, Cat. No. H1399). Imaging and analysis were performed using a BioTek Synergy LX Multimode Reader (Agilent), an Olympus® fluorescence microscope with CellSens® software, a Keyence BZ‐X810 fluorescence microscope, and ImageJ software (NIH). Dynamic culture was achieved using the OrganoFlow® perfusion rocker (MIMETAS). The following reagents and instruments were used for nucleic acid extraction and qPCR analyses: TRIzol reagent (Cat. No. 15596026), SuperScript IV VILO cDNA Synthesis Kit (Cat. No. 11756050), and PowerTrack SYBR Green Master Mix (Cat. No. A46109) (Thermo Fisher Scientific). Quantitative PCR measurements were obtained using the QuantStudio 3 Real‐Time PCR System (Thermo Fisher Scientific, Cat. No. A28137).

### 2.2 Cell culture and spheroid formation

MDA‐MB‐231 cells were maintained as adherent cultures in complete DMEM and kept in a humidified incubator at 37°C and 5% CO_2_, with medium exchanges performed every second day. When cultures reached approximately 85%–90% confluency, spheroid formation was done employing the liquid‐overlay technique in 96 and 384 ULA plates. Their special non‐adherent treatment allows cells to aggregate together. To generate uniform and compact spheroids, 3% (v/v) Matrigel was added at seeding. Plates were centrifuged for 3 min at 250 × *g* and incubated under standard conditions. Half‐medium exchanges were performed every 2 days. MCF‐7 cells were cultured in complete Advanced RPMI 1640 medium and kept in standard incubating conditions. Once the cultures reached the appropriate confluency, spheroids were generated by seeding 2000 cells per well into 96‐well ultra‐low attachment (ULA) plates.

During seeding, cultures were mixed with 3% (w/v) Matrigel to promote aggregation, followed by centrifugation at 150 × *g* for 5 min. The first medium exchange included 2% Matrigel to further support spheroid formation.

### 2.3 Assessing metabolic activity with CCK‐8

To assess cell viability and cytotoxic effects of anticancer agents, a colorimetric assay was performed using the CCK8, which contains the water‐soluble tetrazolium salt WST‐8.^14^ For eight consecutive days, CCK‐8 was added to spheroids at a 1:10 reagent‐to‐medium ratio and incubated for 4 h. Absorbance at 450 nm was detected using a BioTek Synergy LX system, with measurements obtained in triplicate.

### 2.4 Spheroid viability and characterization

To monitor the viability, spheroids were stained with a LIVE/DEAD™ Viability/Cytotoxicity Kit for mammalian cells, following the manufacturer's instructions. A staining medium solution was prepared with 4.0 μM ethidium homodimer (EthD‐1) and 2.0 μM Calcein‐AM. Live‐cell esterases convert Calcein‐AM to a green fluorescent product, whereas EthD‐1 marks dead cells by binding to DNA and emitting red fluorescence.^15^ On selected days, the culture medium was replaced with the staining solution and incubated for 1–2 h to allow penetration into the spheroids.^16^

Additionally, to assess overall spheroid health and determine suitability for further studies, a series of staining techniques were performed at selected time points to evaluate apoptosis, mitochondrial function and nuclear morphology. A 2.0 μM fluorescent Caspase‐3/7 Green Detection Reagent substrate was used to detect apoptotic activity. Caspase‐3/7 are executioner proteases, and their activation is a hallmark of apoptosis.^17^ Tetramethylrhodamine methyl ester perchlorate staining (TMRM) was used at 250 nM to evaluate mitochondrial membrane potential, an important indicator of early apoptosis and mitochondrial integrity.^18^ Hoechst staining: DNA‐specific fluorescent dye Hoechst 33342 was applied at 5 μg/mL^19^ to visualize nuclear morphology, chromatin condensation, and detect apoptotic bodies.^20^ Following incubation, spheroids were washed twice with PBS. Imaging was performed on an Olympus fluorescence microscope using CellSens® software, and images were processed with ImageJ. Each experiment was carried out with three replicates.

### 2.5 Spheroid transfer to pillar/perfusion system and optimization of transfer

The essential steps for transferring spheroids to a 36PillarPlate are as follows. Pillars are first hydrated in a petri dish containing 500 μL sterile water and placed in a humidified incubator for 30 min. At the same time, medium in the 384DeepWellPlates, used for long‐term pillar plate culture,^21^ was pre‐warmed in the incubator to minimize bubble formation in hydrogels. For static culture, pillar plates were coupled with the 384DeepWellPlates, whereas for dynamic culture, the 36‐perfusion plate was utilized and placed on the OrganoFlow® perfusion rocker (MIMETAS) at a 10° tilt with 1‐min intervals.

Spheroids were initially seeded at a higher density (5000 cells/well) to reduce viability loss and improve transfer success, particularly for early invasion studies using Matrigel embedding. However, subsequent transfers used a lower density (2000 cells/well) to minimize diffusion limitations within larger spheroids.^22^, ^23^ For optimization, pillars were coated with varying concentrations of multiple hydrogels. Whole‐plate imaging was performed using a Keyence BZ‐X810 fluorescence microscope, enabling high‐resolution stitched images.

For Matrigel embedding, pillars were coated with 75% (6.7 mg/mL) Matrigel diluted in growth medium to maintain physiological pH.^24^ After manually coating, plates were incubated 1–2 min at room temperature before sandwiching them onto the 384 ULA plates and quickly inverted to allow gravity‐assisted spheroid transfer. Sandwiched plates were transferred to the humidified incubator for 45 min to allow Matrigel gelation.

For alternative embedding, a hybrid hydrogel mixture of Matrigel (50% v/v) and alginate (0.75% w/v) was prepared. Before transfer, a 2 mM CaCl_2_ solution was added to the ULA plates to allow the partial gelation of alginate during transfer.^25^ Pillars were manually coated with the hybrid solution and left to partially gelate for 2–3 min at room temperature. Afterward, the pillar and ULA plates were sandwiched, inverted, and placed in the humidified incubator for 45 min. After incubation, complete alginate gelation is achieved by dipping the pillar plates in a 25 mM CaCl_2_ solution for 5–10 min at room temperature.^26^ Fmoc‐FF‐OH hydrogels were also tested on pillar plates. A 20 mM stock solution was prepared by dissolving the Fmoc‐phe‐phe‐OH powder in sterile water and NaOH (to allow the powder to dissolve more efficiently). Fmoc‐FF has a carboxyl (—COOH) group that requires NaOH for dissolution.^27^ Furthermore, the pH was set to 7 to ensure a physiologically neutral condition suitable for cell growth and gelation condition.^28^ To coat the pillars, the stocks were further diluted to 15, 10, and 5 mM concentrations. Manual dispensing was employed to test multiple concentrations in a single pillar plate. After coating, pillar plates were immediately sandwiched onto ULA plates and kept in the humidified incubator for 30 min to finalize gel stabilization.

Different alginate concentrations of 0.75%, 1%, 1.5%, and 1.75% (w/v) were also evaluated to optimize the spheroid transfer. On the day of transfer, a 2 and 3 mM CaCl_2_ medium solutions (in sterilized water) were prepared to induce partial pre‐gelation in the ULA plates. Alginate concentrations are centrifuged to remove bubbles and carefully dispensed onto the loading plate for stamping. Once pillars are coated, they are immediately sandwiched with the ULA plate, followed by 50 min in the humidified incubator to allow the spheroids perfuse the layer of different alginate concentrations. Lastly, pillars were dipped in a 25 mM medium CaCl_2_ solution for 10 min at room temperature to fully gelate alginate.

Finally, a gelatin–alginate mixture was employed as the selected hydrogel composition for transfer. Alginate and gelatin stock solutions were diluted with distilled water and combined at final concentrations of 1% and 2% (w/v), respectively. The resulting solution was spun and dispensed onto the loading plate for pillar coating via stamping. Once coated, pillars were kept at 4°C for 20 min to allow gelatin to solidify. Pillars were then sandwiched with the ULA plates and placed in the incubator at 37°C for 10 min. Following incubation, pillars were placed in a 25 mM CaCl_2_ medium solution for 5 min to achieve complete alginate gelation.

### 2.6 EISAP peptide treatment

On day 3 of spheroid culture, spheroids with consistent morphology (uniform sphericity, diameters 350–420 μm) were selected for treatment with EISAP (Table 1), as these parameters are critical for reproducibility in drug testing.^22^

**TABLE 1:** Molecular structure and molecular weights of peptide constructs determined by mass spectrometry.

Peptide	Observed MW (*m*/*z*)
P1: Fmoc‐FF‐pTyr	776.2
P2: Fmoc‐FF‐pThr	714.2
P3: RGD‐FF‐pTyr	884.8
P4: NBD‐FF‐pTyr	772.0
P5: Nap‐FF‐pTyr	722.2
P6: Nap‐FF‐pThr	662.2

Treatment was carried out under static (384DeepWellPlate) and dynamic conditions (36‐perfusion plate and OrganoFlow® rocker at 10° tilt in 1 min intervals). Spheroids were initially exposed for 72 h to four different concentrations (10, 50, 100, and 200 μM) of the self‐assembling peptide P1 (Fmoc‐FF‐pTyr), a compound that undergoes enzyme‐instructed self‐assembly in tumor environments.^29^ After assessing the viability of spheroids treated for 72 h with P1 alone, the same concentrations of P1 were co‐administered with 5 μM DXR: Doxorubicin hydrochloride, a standard chemotherapeutic agent, to evaluate potential boosting effects for 48 h.^30^

Each condition was tested in triplicates under standard incubation conditions. To quantify viability, the CCK‐8 assay was performed at selected time points.

For gene expression analysis, a pool of 50–60 spheroids was treated in the ULA plates with a panel of peptides including Fmoc‐FF‐pTyr (P1), Fmoc‐FF‐pThr (P2), RGD‐FF‐pTyr (P3), Nap‐FF‐pTyr (P5), and Nap‐FF‐pThr (P6) at 200 μM for 72 h under static and standard incubation conditions. All peptides were dissolved in PBS with pH 7.

### 2.7 Imaging distribution of NBD‐FF‐pTyr with fluorescence microscope

For peptide distribution visualization, spheroids were incubated (37°C, 5% CO_2_) with 250 μM of the self‐assembling NBD‐Phe‐Phe‐pTyr (P4) for 5 days. Daily imaging was performed to track peptide spatial distribution within the spheroids using Olympus® fluorescence microscopy equipped with CellSens® imaging software.^31^

To minimize background fluorescence and enhance image and signal clarity, spheroids required a minimum of two PBS washes before imaging. Relative Fluorescence Units (RFU) for quantitative measurements were obtained with the BioTek Synergy LX Multimode Reader.

### 2.8 Gene expression analysis with qRT‐PCR

Total RNA was extracted from the treated spheroids in the ULA using TRIzol. Using 1 μg of RNA, complementary DNA (cDNA) was synthesized with the SuperScript IV VILO Master Mix Kit following the supplier's protocol. Quantitative PCR was carried out with Power‐Track SYBR Green Master Mix on the QuantStudio 3 system using gene‐specific primers (Table 2). Data were processed using the ∆∆*Ct* approach, and expression values were normalized to the reference gene *GAPDH*.

**TABLE 2:** qRT‐PCR primer sequences used for gene expression analysis.

Gene name	Forward primer (5′–3′)	Reverse primer (5′–3′)
*BCL2*	GAGGAGCTCTTCAGGGACGG	GTTCCACAAAAGGCATCCCAGC
*BRCA2*	CTCGGGTGTCTTTTGGCGC	TGCTTGATAAATAGTCCGCTCC
*TP53*	GCAGTCACAGCACATGACGG	AGTCAGGCCAAACCTCAGGC
*GAPDH*	GTCTCCTCTGACTTCAACAGCG	ACCACCCTGTTGCTGTAGCAA

### 2.9 Statistical analysis

Quantitative data are expressed as mean values ± with standard deviation (SD) based on three independent replicates. Pairwise group comparisons were analyzed using Student's *t*‐test, whereas multiple comparisons were evaluated using two‐way ANOVA with Tukey's post hoc correction. Statistical significance was defined at *p* < 0.05 (*) or *p* < 0.01 (**).

## 3 RESULTS AND DISCUSSION

### 3.1 Establishing 3D spheroids in ULA's for EISAP testing

To establish a robust 3D model for evaluating EISAPs under physiological relevant models, MDA‐MB‐231 and MCF7 spheroids were cultured in ULA plates over the course of 14 days and systematically evaluated for several critical parameters during the characterization phase, including (i) spheroid size and density, (ii) metabolic performance, and (iii) viability within the aggregate. These baseline evaluations provided a reference for spheroid growth and function before subsequent transfer to pillar plates for hydrogel optimization, ensuring that the model accurately mimics in vivo TME, known to influence drug response and cellular behavior.^22^, ^32^

**FIGURE 1:**
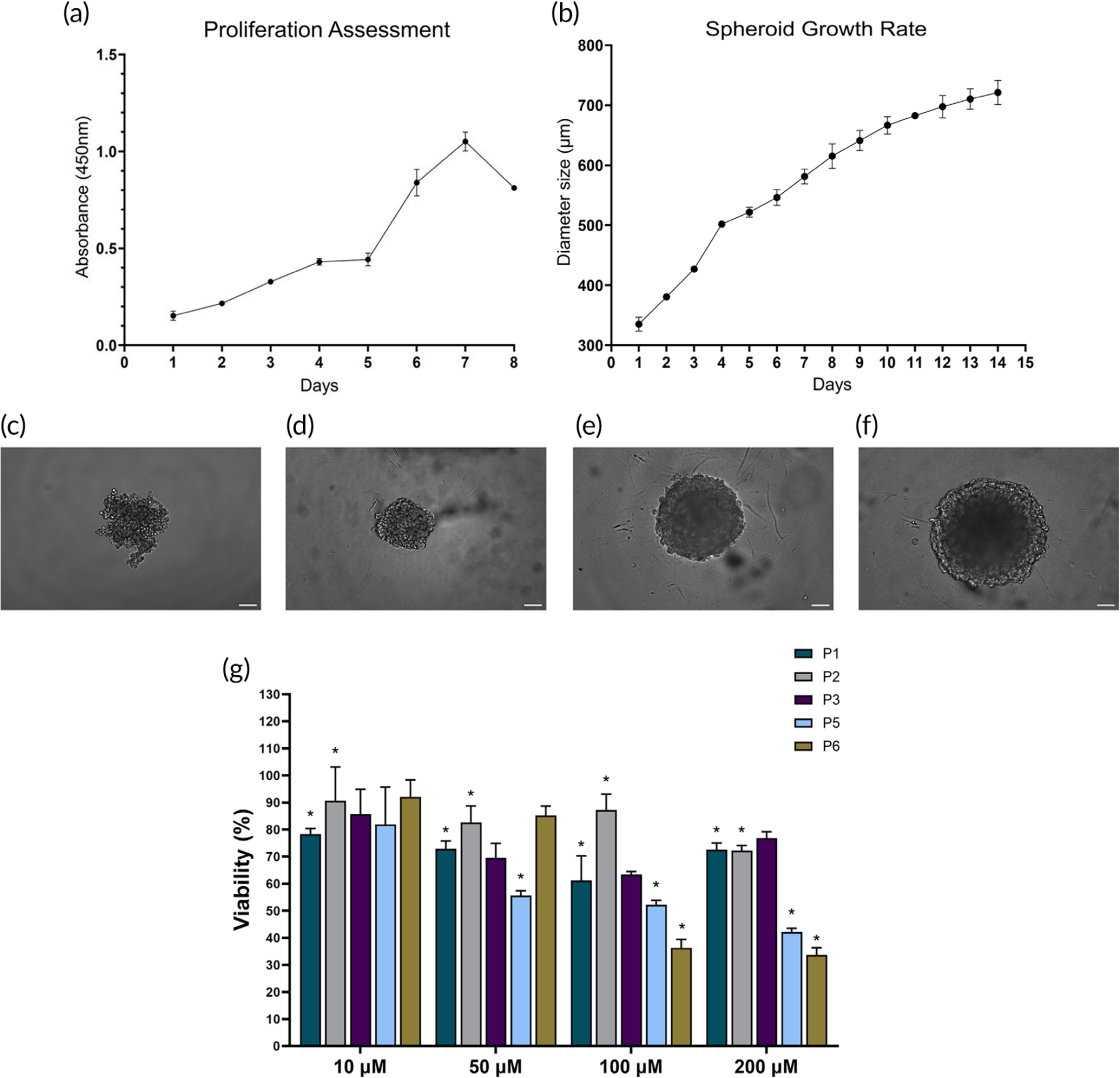
MDA‐MB‐231 spheroids cultured in ULA plates. (a) Cell proliferation over 8 days assessed using the CCK‐8 assay. (b) Spheroid size monitored for 14 days. (c) Spheroid morphology on day 2 without Matrigel. (d) Spheroid morphology on day 2 with 3% Matrigel. (e) Spheroid appearance on day 4. (f) Spheroid morphology on day 8 showing progressive growth. (g) Viability of TNBC spheroids treated with five EISAPs at ranging concentrations of 10–200 μM, quantified using the CCK‐8 assay. Data represent mean *±* SD of triplicates. Asterisks indicate differences relative to untreated controls (*p < 0.05). Scale bars = 100 μM.

Metabolic activity was monitored daily from day 0 to day 8 using the CCK‐8 assay. A progressive rise was detected beginning at day 1, reaching its peak on day 7 before declining sharply on day 8 (Figure 1a). This trend reflects active proliferation during the early phase, with reduced activity indicating stress, nutrient depletion, or maturation.^33^ Based on these observations, days 3–7 were selected as the optimal testing window. Such trends align with previous studies showing that TNBC spheroids maintain stable diameter and high metabolic activity during the first 9 days of culture, depending on initial seeding density.^22^, ^32^

Spheroid size was tracked over 14 days. A steady increase in diameter was observed from day 1, with a marked spike on day 4 (Figure 1b). It is well established that spheroids larger than 500 μm develop necrotic cores due to oxygen and nutrient diffusion limits, compromising physiological relevance.^34^ Thus, size‐dependent effects must be carefully considered in experimental design. The observed growth dynamics further support days 3–7 as the optimal testing period.

Spheroid formation was strongly influenced by two factors: initial seeding density and ECM incorporation. The addition of 3% (v/v) Matrigel significantly enhanced ECM deposition, resulting in compact, uniform, and structurally stable spheroids, as seen in Figure 1d–f. In particular, spheroids maintained integrity at day 4 (Figure 1e) and continued to show stable growth by day 8 (Figure 1f). This ECM enrichment promoted stronger intercellular and matrix‐associated interactions, both essential for spheroid integrity and TME mimicry.^24^ In contrast, spheroids formed without Matrigel showed looser, heterogeneous aggregation (Figure 1c), reducing physiological relevance and assay reproducibility. For prescreening, MDA‐MB‐231 ULA spheroids were treated with EISAPs on day 3, and viability was assessed after 72 h. The viability of spheroids treated with peptides P1, P2, P3, P5, and P6 evaluated across concentrations ranging from 10 to 200 μM is shown in (Figure 1g). All peptides reduced viability in a dose‐dependent manner, with P5 and P6 showing the strongest cytotoxic effect in MDA‐MB‐231 spheroids, particularly at 200 μM. P1, at its optimal concentration of 100 μM, reduced spheroid viability to 58%.

**FIGURE 2:**
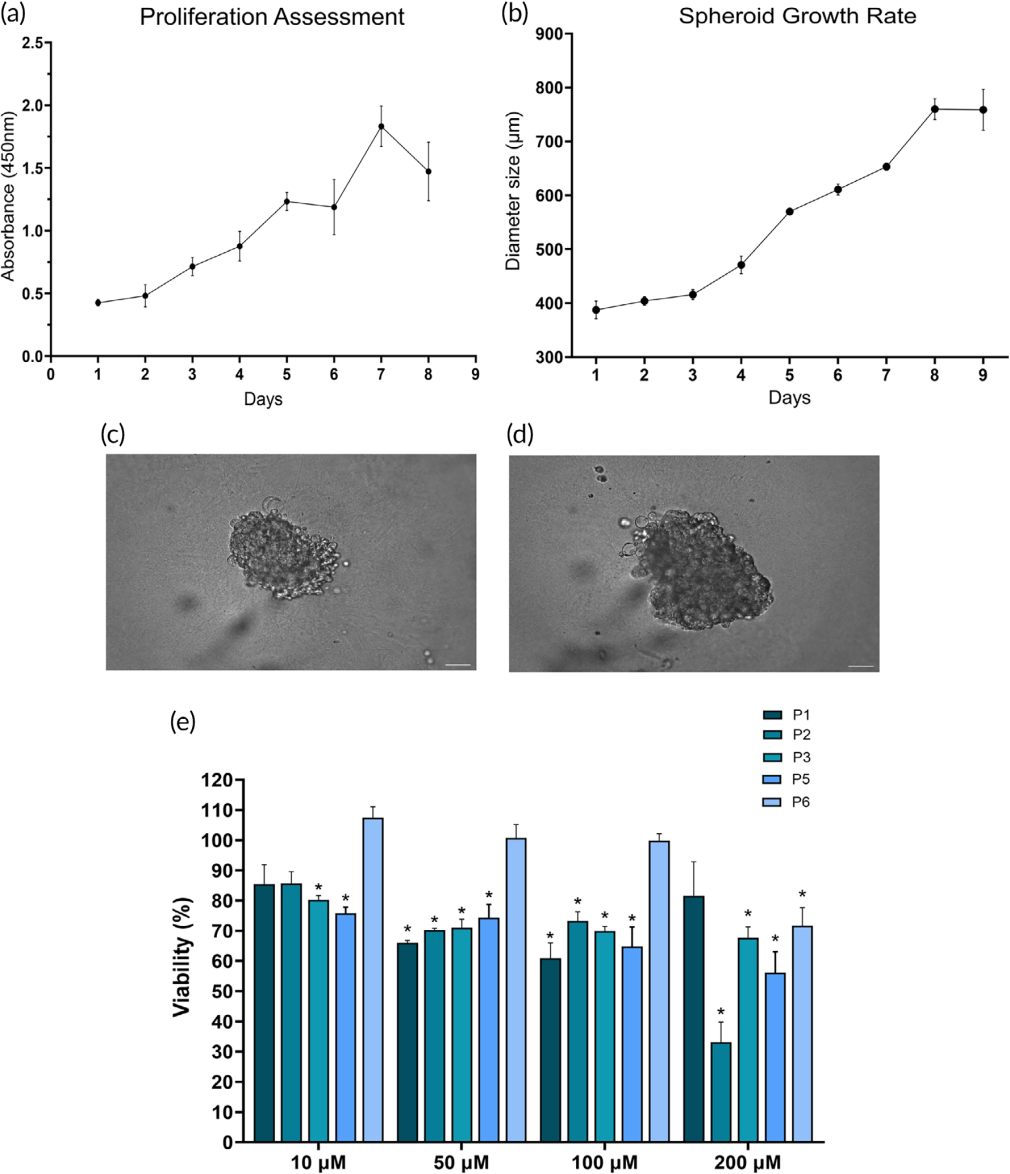
MCF‐7 spheroids cultured in ULA plates. (a) Cell proliferation over 8 days assessed using the CCK‐8 assay. (b) Spheroid size monitored for 9 days of culture. (c) Spheroid morphology on day 2. (d) Spheroid morphology on day 4 showing progressive growth. (e) Viability of MCF‐7 spheroids treated with five EISAPs at ranging concentrations of 10–200 μM, quantified using the CCK‐8 assay. Data represent mean *±* SD of triplicates. Asterisks indicate significance relative to untreated controls (*p < 0.05). Scale bars = 100 μm.

Proliferation of MCF‐7 cells in spheroids was measured over 8 days and shows a steady increase in metabolic activity, indicating continuous cell growth (Figure 2a). Growth kinetics of MCF‐7 spheroids demonstrate a progressive increase in spheroid diameter from day 1 to day 9, confirming robust 3D culture expansion (Figure 2b). Representative bright‐field images of MCF‐7 spheroids at day 2 and day 4 illustrate increasing spheroid compaction and enlargement over time (Figure 2c,d). Figure 2e indicates a concentration‐dependent decrease in MCF‐7 spheroid viability across peptides P1–P6. At 100 μM, P1 produces the most pronounced reduction in viability within its dose range, indicating higher activity at this concentration compared to the other peptides. At 200 μM, P2 and P5 show the greatest decrease in spheroid viability, demonstrating their maximal effectiveness at the highest tested dose. Overall, the results highlight distinct dose–response profiles for each peptide, with P1, P2, and P5 showing the highest effectiveness at their respective concentrations. Compared with MCF‐7 spheroids, those formed from MDA‐MB‐231 cells exhibited a more rounded morphology and developed into a more robust, reproducible 3D model.

### 3.2 Characterization of spheroid viability and microenvironment features

**FIGURE 3:**
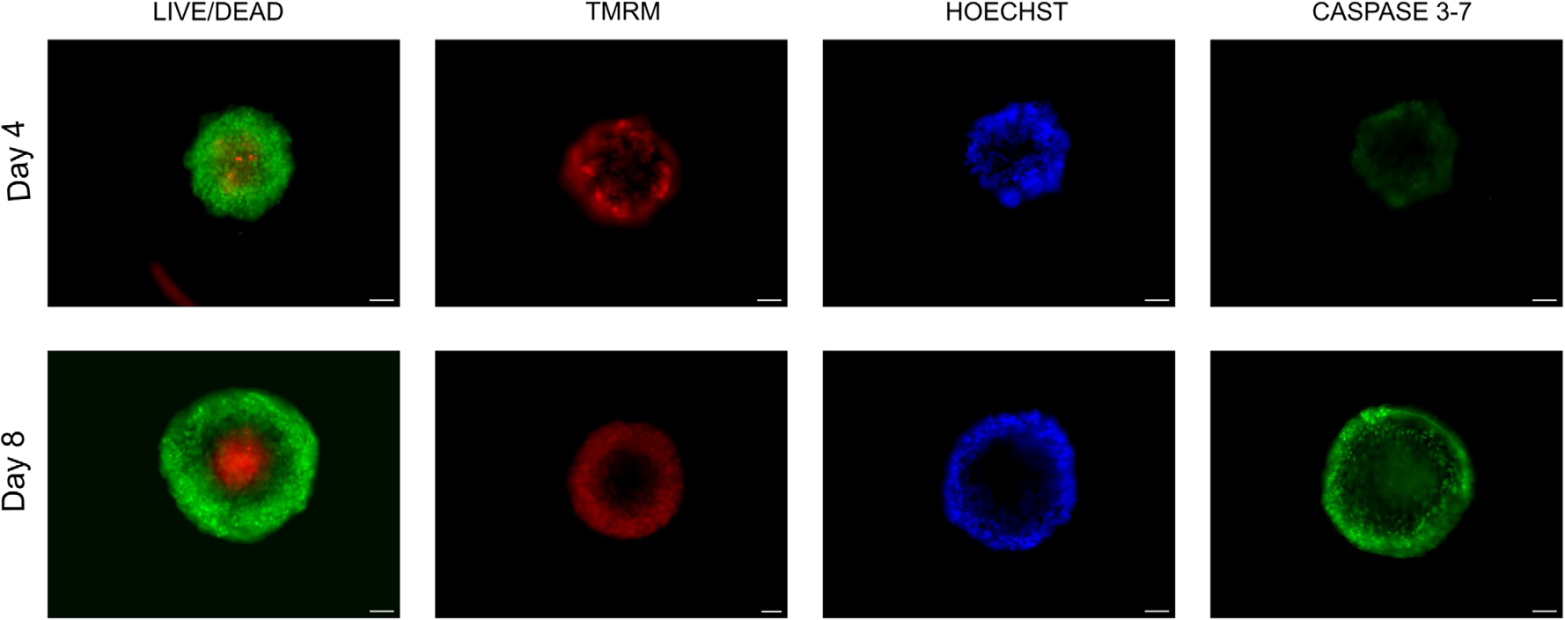
Fluorescence images characterizing spheroid viability (MDA‐MB‐231) and microenvironment features on day 4 and 8, LIVE/DEAD staining displays a highly viable spheroid at day 4 and increased necrotic core by day 8. TMRM mitochondria staining shows strong mitochondrial activity in the periphery compared to the core at day 8. Hoechst DNA staining indicates intact nuclear morphology at both time points. Caspase 3–7 activity was minimal at day 4 but increased at day 8, consistent with apoptosis progression. Scale bars = 100 μM.

To assess spheroid health, metabolic activity, and apoptosis over time, a series of staining assays were performed on MDA‐MB‐231 spheroids (Figure 3). LIVE/DEAD staining at days 4 and 8 revealed a higher proportion of viable cells at earlier time points, with increasing cell death localized to the spheroid core by day 8. This pattern is consistent with the diffusion‐limited microenvironment of spheroids in ULA plates, where central depletion of oxygen and nutrients leads to hypoxia and necrosis.^35^

Mitochondrial membrane potential was measured using the fluorescent probe TMRM (tetramethylrhodamine methyl ester perchlorate). Strong mitochondrial activity was observed at the spheroid periphery at both time points, indicating higher metabolic activity in outer cells compared to the hypoxic core, a hallmark of large spheroids.^23^ Hoechst staining showed a relatively uniform nuclear distribution with intact morphology on day 4, reflecting preserved cellular organization during early culture. By day 8, nuclear morphology remained intact, though apoptotic activity increased.

Apoptotic signaling was further analyzed using Caspase‐3/7 staining. Minimal signal was detected in early stages, primarily in the core, but by day 8, Caspase‐3/7 activity increased significantly, suggesting progression toward apoptosis and potential secondary necrosis in response to hypoxic stress.^36^ Collectively, these results indicate that spheroids are more viable and metabolically active at early stages (days 3–5), while reduced viability and increased apoptosis dominate on day 8. Thus, early‐stage spheroids represent a more suitable window for therapeutic testing and mechanistic studies.

### 3.3 Hydrogel optimization for 3D spheroid embedding in pillar plate platforms

**FIGURE 4:**
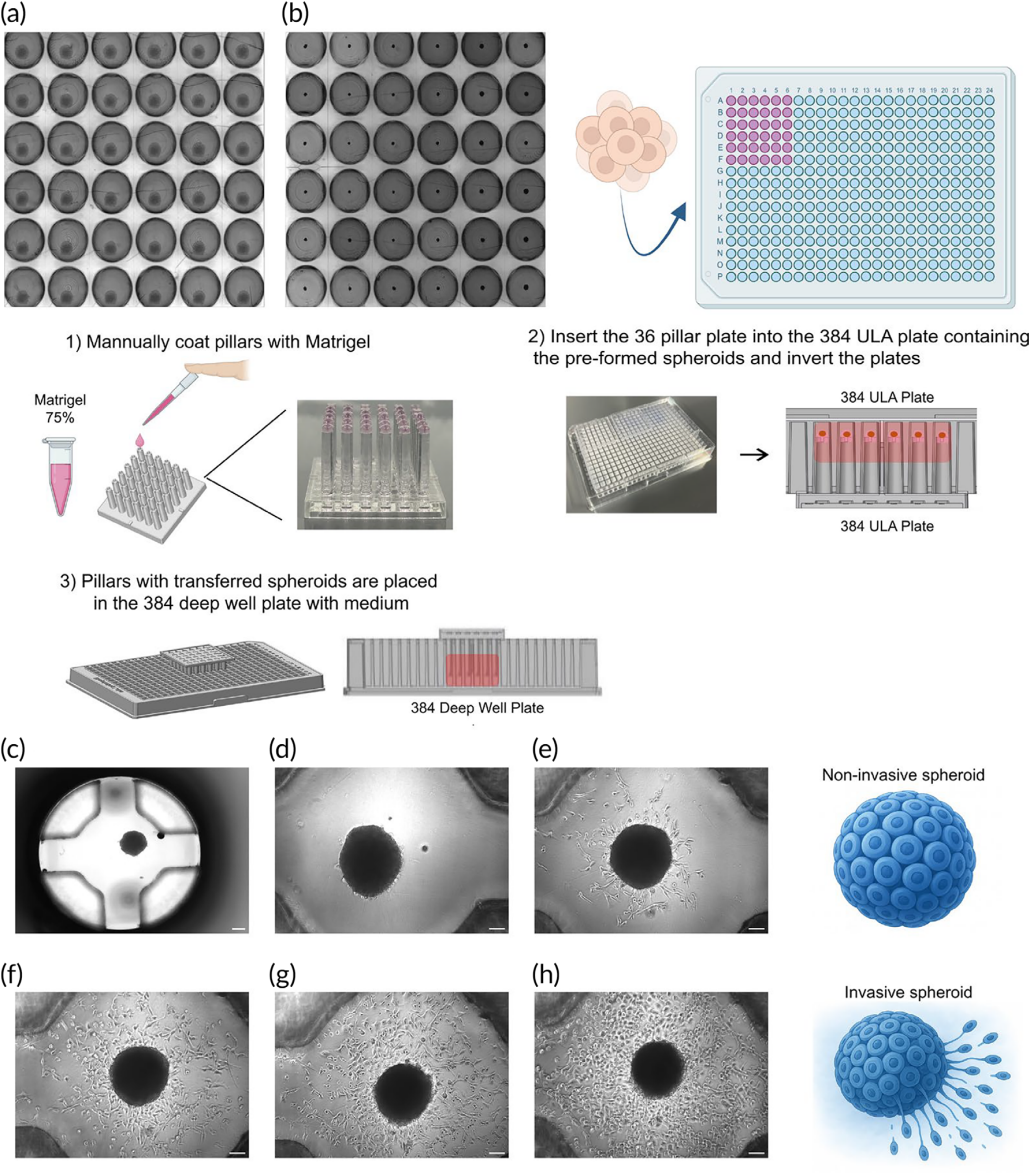
(a) Stitched image of 384 ULA after cell seeding and centrifugation. (b) Stitched image of ULA plate showing successful aggregation and compact spheroid formation before transfer. (c) 4× image of spheroid after transfer on a pillar with 75% Matrigel. (d) 10× image of spheroid 8 h post‐transfer. (e) 24 h post‐transfer. (f) 48 h post‐transfer. (g) 72 h post‐transfer. (h) 96 h post‐transfer (scale bars = 100 μm). (1–3) Schematic of transferring spheroids from ULA to 36PillarPlate.

We utilized a pillar–perfusion plate system to culture 3D spheroids directly on hydrogel‐embedded pillars.^21^ The pillar plate was optimized for MDA‐MB‐231 cells, as these cells exhibited a more rounded morphology compared to MCF‐7. The pillar–perfusion platform supports 3D structures cultured on individual pillars, where sidewalls and slits allow spheroid encapsulation in hydrogel, enabling both static and dynamic culture conditions. Dynamic media flow is achieved by pairing the pillar plate with the perfusion plate and operating them on a digital rocker that provides bidirectional flow through adjustable tilt angles. This configuration facilitates direct loading of breast cancer spheroids onto pillars, streamlining handling, media exchange, and treatment application.^21^, ^37^ To optimize spheroid transfer from ULA plates to the pillar plate, we tested multiple hydrogel formulations, including Matrigel, alginate, alginate–Matrigel, gelatin–alginate, and Fmoc‐FF‐OH (Table 3). Following transfer, the pillar plate was immersed in a companion deep‐well plate to ensure that spheroids remained fully submerged in culture medium and viable throughout treatment and handling. Matrigel was initially used to improve spheroid embedding and transfer efficiency. MDA‐MB‐231 spheroids embedded in Matrigel displayed invasive outgrowth, particularly at higher seeding densities. Pillars coated with 75% (6.5 mg/mL) Matrigel and seeded with either 2000 or 5000 cells per spheroid achieved a transfer efficiency of 91%. However, spheroids seeded with 5000 cells showed extensive invasion within 2 days of transfer (Figure 4), whereas those seeded with 2000 cells maintained greater structural integrity and exhibited slower, reduced invasion. This suggests that lower initial seeding densities help preserve spheroid structure and delay invasive behavior.

**TABLE 3:** Summary of spheroid behavior under multiple hydrogel conditions.

Conditions	Transfer rate	Detachment	Invasion
Matrigel 75%	91.6%	✘	✔
Matrigel 50% and alginate 0.75%	80.5%	✘	✔
Alginate‐2 and 3 mM (0.75%, 1%, 1.5%, 1.75%)	33.3%, 11.1%, 0%, 33.3% 11.1%, 11.1%, 44.4%, 0%	✘	✘
Fmoc‐Phe‐Phe‐OH (5, 10, 15, and 20 mM)	11.1%, 16.6%, 33.3%, 11.1%	✔	✘
Alginate 1% and gelatin 2%	91.6%	✘	✘

*Note*: ✔ = observed; ✘ = not observed.

**FIGURE 5:**
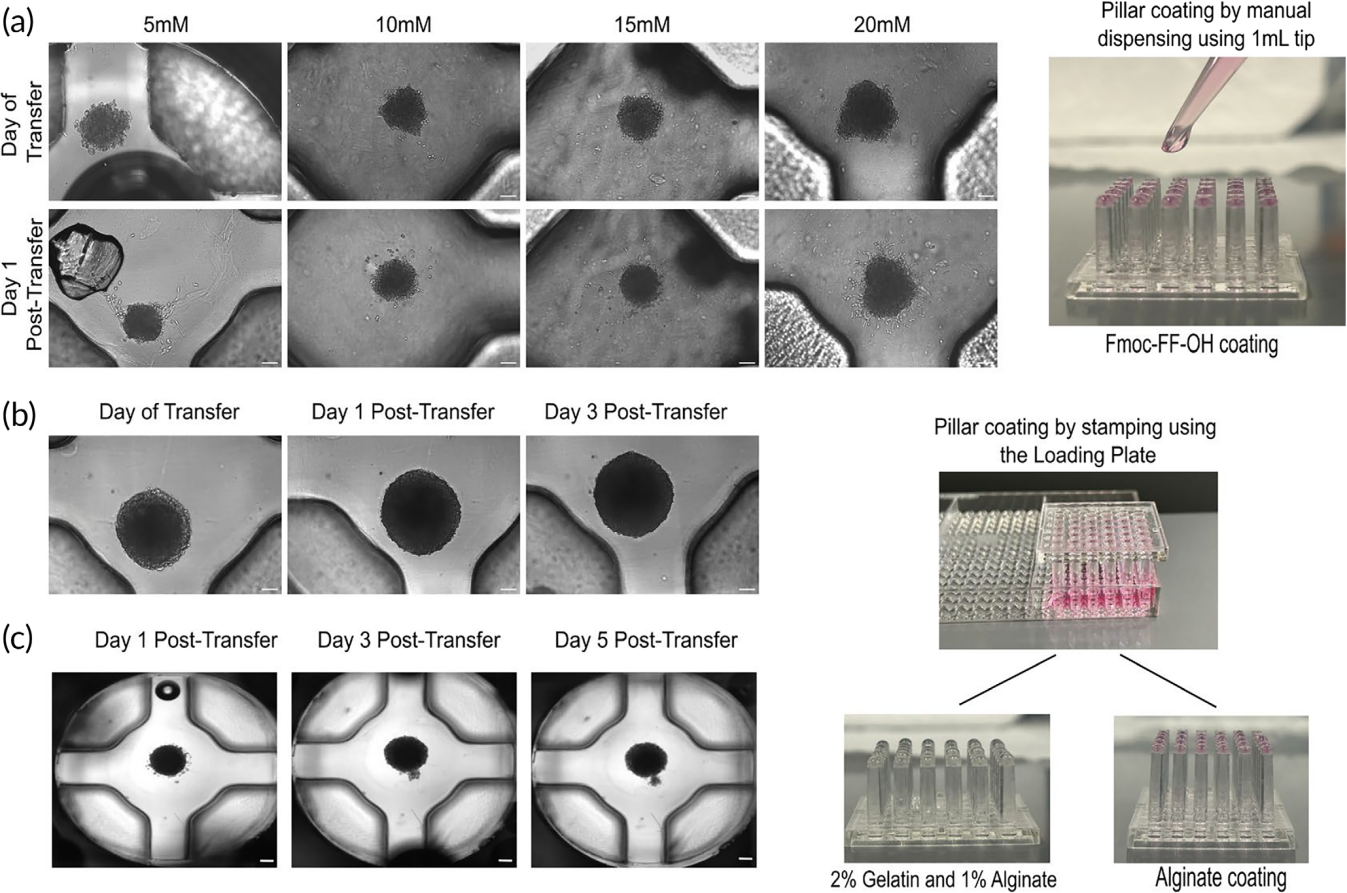
Representative images from the hydrogel optimization process for spheroid transfer. (a) Fmoc‐Phe‐Phe‐OH hydrogels at 5, 10, 15, and 20 mM, imaged immediately after transfer (day 0) and on day 1 post‐transfer. Imaging was limited to the first 2 days due to major spheroid detachment observed beyond day 1. Scale bars = 100 μm. (b) Pillars coated with alginate‐alone: Spheroid transfer with 1.5% (w/v) alginate pre‐gelled with 3 mM CaCl_2_. Scale bars = 100 μm. (c) Optimized transfer condition: 1% (w/v) alginate and 2% (w/v) gelatin, yielding the most stable and intact spheroids post‐transfer. Scale bars = 200 μm.

We next investigated Fmoc‐FF‐OH gels for transfer success and viability (Figure 5). In our prior work, we characterized the rheological properties of Fmoc‐FF at different concentrations and found that 2% and 5% gels with G′ of 100 and 1000 Pa preserved cell viability.^38^

Based on their self‐assembly and ECM‐mimicking properties,^27^ we tested concentrations of 5, 10, 15, and 20 mM. The highest success rate (33.3%) occurred at 15 mM, followed by 16.6% at 10 mM. However, spheroids detached within 48 h regardless of concentration, possibly caused by insufficient mechanical stability of the soft, viscoelastic gel (Figure 5a).

Alginate was also evaluated across concentrations (0.75%–1.75%, w/v) and crosslinking conditions with CaCl_2_ (2 and 3 mM) to assess mechanical stability and spheroid retention. Higher cell density (5000 cells/well) improved transfer, likely due to better and faster penetration into the hydrogel. With 2 mM CaCl_2_, success rates were low (0%–33.3%), but 3 mM improved transfer, particularly at 1.5% (w/v) alginate (44.4%). Despite initial success, spheroid darkening and necrotic core formation were observed, as a result of high seeding density and limited nutrient/oxygen diffusion through alginate (Figure 5b). To improve this, alginate was combined with Matrigel at a concentrations of 0.75% (w/v) and 50% (v/v), respectively. Invasion persisted due to Matrigel's intrinsic bioactivity, but progressed in a more confined and robust manner.

To further improve hydrogel stability and ensure spheroid attachment, gelatin was incorporated into the alginate formulation. Gelatin enhances cell–ECM interactions and increases the viscoelastic properties of alginate‐based hydrogels, making them more suitable for dynamic culture conditions.^39^ An optimized 2% gelatin and 1% alginate mixture was used to coat the pillars, resulting in a 91.6% spheroid transfer rate, with no detachment observed during handling or perfusion. Spheroids maintained their structural integrity and viability over time and remained compact and non‐invasive, unlike those cultured in Matrigel–alginate (Figure 5c). Altogether, these observations demonstrate that the gelatin–alginate coating offers robust mechanical support and biocompatibility for long‐term dynamic culture, validating its use as the optimal coating for subsequent experiments.

### 3.4 3D invasive spheroid response to Co‐treatment in pillar plates

As previously discussed, we identified that coating the pillars with Matrigel supported the development of an invasive spheroid model during hydrogel optimization. This environment recreated conditions favorable for cancer cell invasion, and the pillar plate system provided a consistent and reproducible platform for downstream experiments. Recognizing this behavior, we leveraged Matrigel‐based cultures as a comparative model to evaluate the effects of EISAPs and DXR in both invasive and non‐invasive settings.

Cancer metastasis takes place when cancer cells propagate to distant tissues and trigger the establishment of secondary tumors. This process occurs in what is known as metastatic cascade, involving multiple steps such as local invasion, intravasation into the vasculature, circulation, extravasation into distant tissues, and secondary tumor establishment.^40^ Invasion involves the loss of cell–cell adhesion, enabling cancer cells to detach and infiltrate surrounding tissue. This step is facilitated by degradation of the ECM and basement membrane, alongside changes in proteins regulating motility and migration.^41^

TNBC cells are highly aggressive and their progression is associated with the epithelial‐to‐mesenchymal transition (EMT), a process that equips cancer cells with migratory and invasive properties.^42^ Ding et al. reported that MDA‐MB‐231 spheroids exhibit pronounced EMT profiles relative to 2D cultures, with suppressed E‐cadherin, elevated N‐cadherin, and greater invasive capacity.^43^ This behavior is linked to altered cell–matrix interactions and adhesion molecule dynamics: E‐cadherin supports cell–cell adhesion, whereas N‐cadherin promotes motility and invasion. Consequently, these molecular changes collectively facilitate detachment and migration through the ECM.

The ECM composition surrounding spheroids strongly influences invasion. Basement membrane extracts such as Matrigel contain collagen, entactin, laminin, and growth factors,^44^ mimicking the in vivo basal lamina that carcinoma cells must penetrate during local invasion.^45^ Laminin and collagen IV in Matrigel provide ligands for integrins, activating adhesion pathways essential for invasion.^46^

**FIGURE 6:**
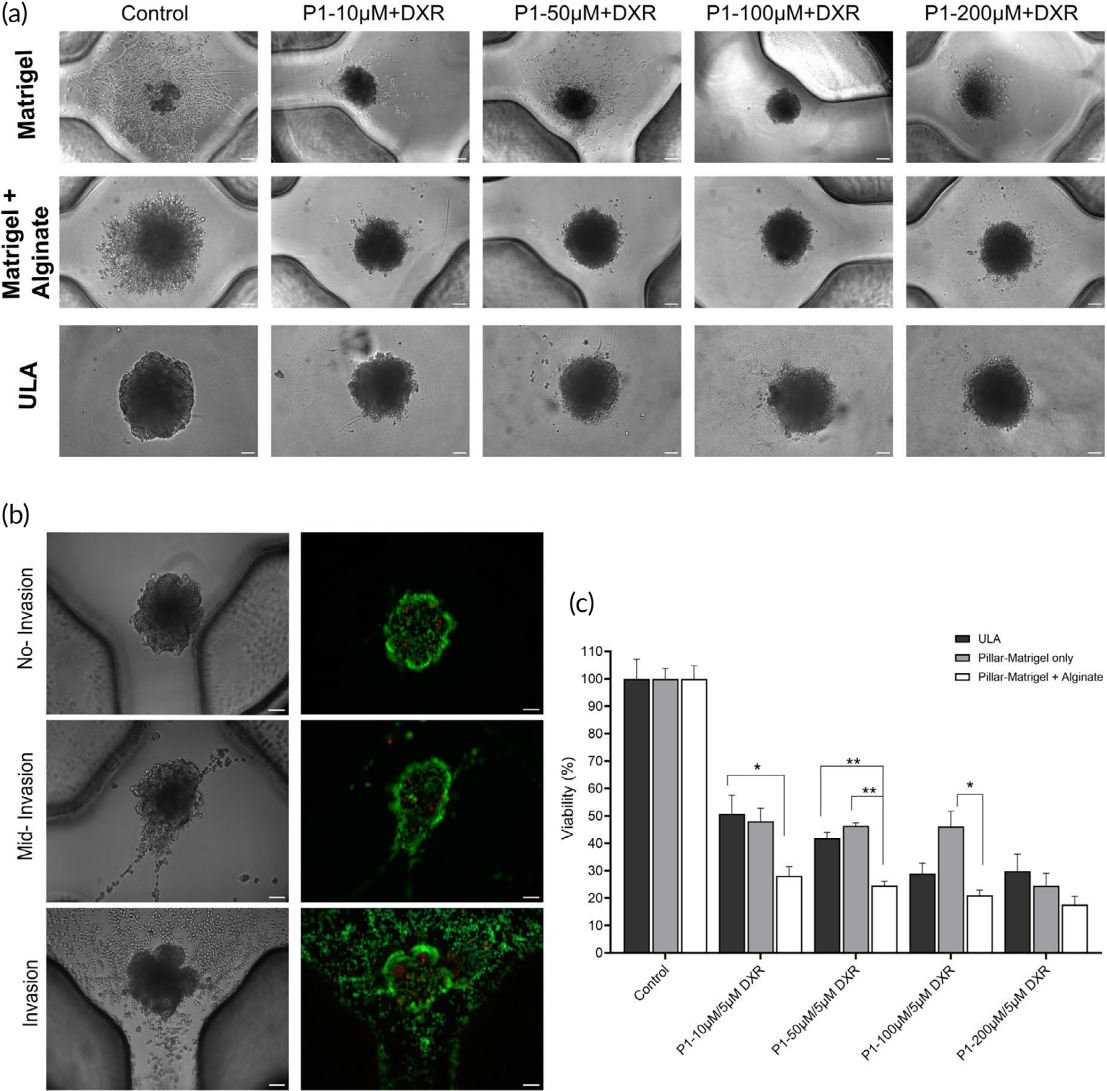
(a) Bright‐field images of MDA‐MB‐231 spheroids co‐treated with increasing concentrations of P1 and DXR under different embedding conditions. In Matrigel, invasive outgrowth was progressively suppressed at higher concentrations, whereas Matrigel–alginate conditions showed a confined morphology with complete inhibition of invasion at higher P1/DXR doses. Spheroids in ULA plates exhibited a smooth, non‐invasive morphology with reduced density at higher P1/DXR concentrations (scale bar = 100 μm). (b) Representative bright‐field and corresponding LIVE/DEAD fluorescence images showing three stages of invasion (no invasion, mid‐invasion, and extensive invasion) in Matrigel spheroids stained with Calcein AM (green, live) and ethidium homodimer‐1 (red, dead) (scale bar = 100 μm). (c) Spheroid viability in ULA, Pillar–75% Matrigel, and Pillar–50% Matrigel with 0.75% alginate after 72 h treatment with P1 and 5 μM DXR. Data are presented as mean *±* SD of triplicates. Statistical significance was evaluated using a two‐way ANOVA to assess the effects of embedding system and peptide concentration, followed by Tukey's post hoc multiple comparison test. Asterisks indicate significant differences relative to the untreated control (*p < 0.05, **p < 0.01).

To generate a robuster model, we refined the Matrigel formulation by incorporating 0.75% alginate. Invasion persisted due to the 50% Matrigel concentration, but its progression was more confined, producing a strong, flower‐like invasive morphology (Figure 6a). To evaluate treatment effects under these conditions, we tested co‐treatment with P1 and DXR across three systems: Matrigel alone, Matrigel–alginate, and ULA (non‐embedding, medium‐only control). Interestingly, in the Matrigel–alginate condition, co‐treatment with EISAP and DXR markedly reduced spheroid viability and completely suppressed invasive outgrowth beyond spheroid boundaries (Figure 6a). The progression of invasion in Matrigel‐based hydrogels was monitored using bright‐field imaging and LIVE/DEAD staining (Figure 6b). Non‐invasive spheroids retained smooth contours and compact structures. At mid‐invasion, cells migrated outward, forming viable protrusions. In fully invasive spheroids, dispersed single cells were observed around the periphery, with high viability confirmed by fluorescence imaging.

As shown in Figure 6c, spheroid viability varied significantly across embedding conditions following P1/DXR co‐treatment, highlighting the impact of culture environment on therapeutic outcome. Matrigel–alginate spheroids consistently showed the lowest viability, Matrigel‐only spheroids the highest, and ULA spheroids intermediate levels. Notably, despite Matrigel concentration being reduced from 75% to 50% in the Matrigel–alginate condition, viability remained lower than in ULA controls, which lack ECM and invasion capacity. This indicates that the effect cannot be attributed solely to Matrigel dilution. Instead, it suggests a synergistic role of alginate confinement and EISAP‐induced cytotoxicity, with the ECM environment directly influencing therapeutic efficacy. In support of this observation, viability in Matrigel–alginate spheroids was significantly reduced compared to both ULA and Matrigel‐only controls at 10, 50, and 100 μM P1/DXR (*p <* 0.05, *p <* 0.01). These results, therefore, demonstrate that the ECM environment critically modulates spheroid response to P1/DXR co‐treatment. The significant lower viability observed in the Matrigel–alginate condition highlights the potential of ECM composition to enhance drug efficacy, likely by affecting drug penetration and cell–ECM interactions. Importantly, Gong et al., who developed composite hydrogels by integrating sodium alginate (SA) into Fmoc‐FF self‐assembled peptide networks, reported that incorporating SA improved hydrogel mechanics and promoted nanowire and honeycomb‐like architectures. The presence of SA reduced the rate of peptide degradation and provided a stable scaffold that was critical for maintaining structural integrity and enhancing cytotoxic efficacy in vitro.^47^ Based on this reported synergy between alginate and Fmoc‐FF assemblies, the use of the Fmoc‐based peptide P1 in our study was particularly important for evaluating therapeutic response in alginate‐embedded matrices within the pillar‐plate system.

### 3.5 Peptide screening and co‐treatment with DXR in static and dynamic settings

To compare treatment efficacy under dynamic versus static conditions, we employed a gelatin–alginate system. Incorporating the alginate–gelatin composite into the pillar plate embedding process allowed efficient spheroid transfer, preserved structural integrity, and supported healthy growth without invasive characteristics. This non‐invasive model served as the baseline for testing EISAPs, eliminating invasion‐related confounding effects. EISAPs are activated by phosphatases such as EYA tyrosine phosphatase, which dephosphorylate tyrosine residues (pTyr).^2^, ^48^ Dephosphorylation reduces hydrophilicity, driving self‐assembly into nanostructures through π–π stacking and hydrophobic interactions, enabling tumor‐selective accumulation.^6^, ^48^ Here, we evaluate their therapeutic potential in physiologically relevant 3D models under dynamic flow conditions, which better mimic the in vivo TME.

**FIGURE 7:**
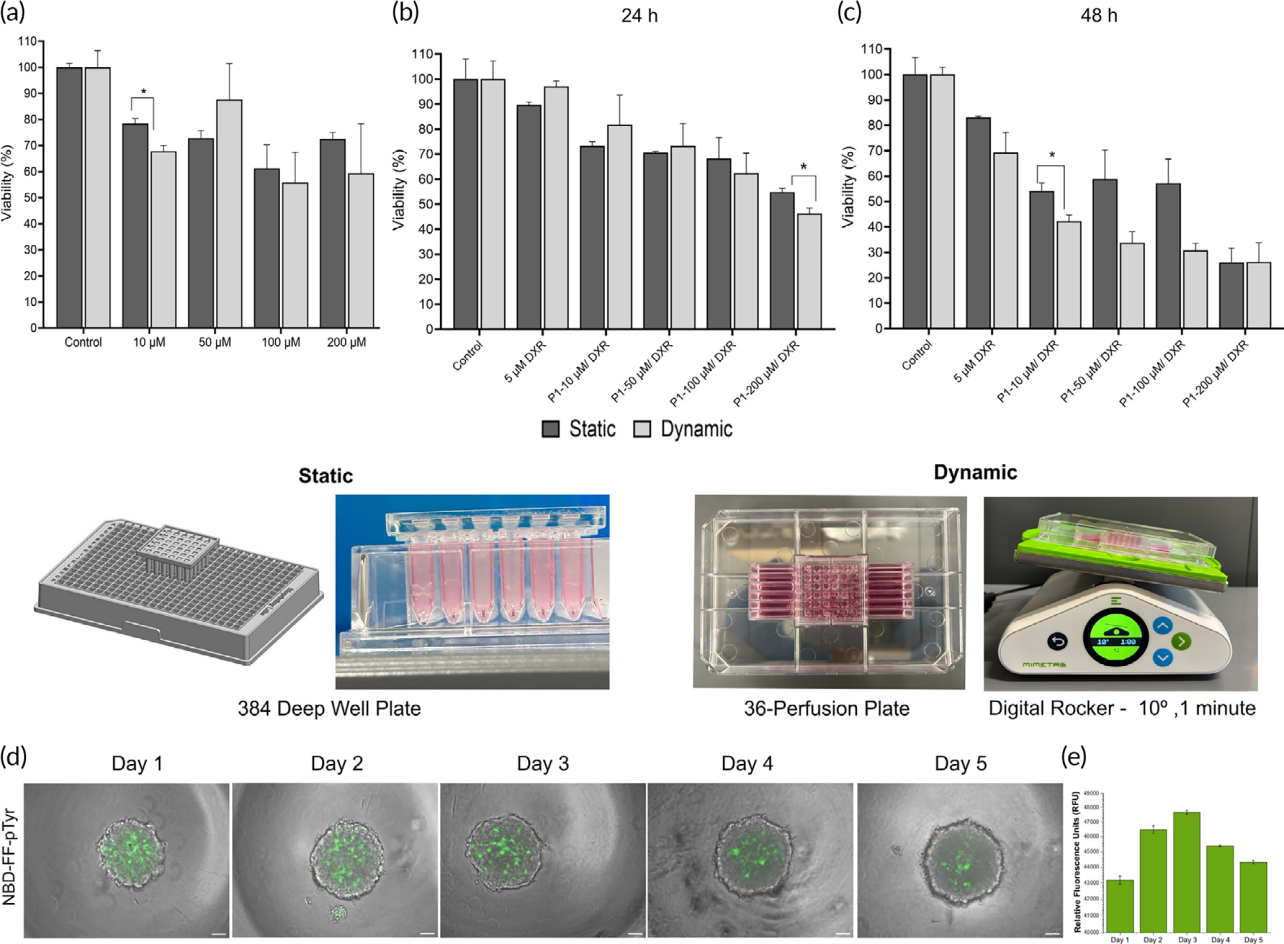
(a) Viability of MDA‐MB‐231 spheroids after treatment with P1 at 10, 50, 100, and 200 μM after 72 h under static and dynamic conditions. (b, c) Viability of MDA‐MB‐231 spheroids after treatment with P1 in combination with 5 μM DXR, under static and dynamic conditions at 24 and 48 h, respectively. *p < 0.05. (d) Representative images showing NBD‐FF‐pTyr distribution over 5 days. Fluorescence was initially localized at the periphery (day 1), progressively penetrated the spheroid core with increased intensity (days 2–3), and slightly decreased thereafter (days 4–5), suggesting redistribution or partial degradation. Scale bars = 100 μm. (e) Quantification of relative fluorescence units (RFU) confirmed these trends, with fluorescence peaking at day 3 before declining. Data represent mean ± SD of triplicates.

We first investigated the performance of P1 in 3D breast cancer spheroids cultured under static and dynamic conditions (Figure 7a). Treatment with P1 reduced viability in both models, with the strongest effect observed at 100 μM. Under dynamic culture, viability dropped to 55%, suggesting that continuous flow enhanced P1 self‐assembly and cytotoxic activity, further confirming the robustness of P1's effects under physiologically relevant conditions.

Next, we assessed whether P1 could enhance DXR efficacy in 3D spheroids. Dynamic flow in the pillar plate system more closely replicates interstitial fluid movement in tumors, promoting nutrient gradients, drug distribution, and ECM remodeling, factors critical for evaluating drug response. Co‐treatment with P1 and 5 μM DXR significantly reduced viability compared to DXR alone (Figure 7b,c). Notably, dynamic culture amplified cytotoxic effects at both 24 and 48 h, likely due to continuous compound perfusion and waste removal, generating gradients and shear forces similar to in vivo conditions.^49^ A clear time‐ and concentration‐dependent viability reduction was observed under dynamic flow, with effects becoming more pronounced at 48 h. Compared to static conditions, dynamic culture consistently exhibited lower viability across all tested concentrations, reflecting enhanced diffusion and compound penetration. After 24 h, viability differences were more evident at higher peptide concentrations, reaching statistical significance at 200 μM P1/DXR, and by 48 h, the difference between systems became more pronounced, underscoring the advantage of perfused platforms in producing more predictive dose–response behaviors.^37^

### 3.6 Imaging distribution of fluorescence‐tagged NBD‐FF‐pTyr in tumorspheroids

Studies have shown that NBD (7‐nitrobenz‐2‐oxo‐1,3‐diazol) is an effective tool to explore peptide internalization and self‐assembly in biological contexts. NBD labeling is employed to track peptide distribution in 3D models, implying active morphological transitions thanks to enzyme‐triggered interactions that allow peptides to go from soluble to β‐sheet nanofibers. Here, we assessed the spatial distribution of peptides in MDA‐MB‐231 spheroids by exposing them to a fluorescence‐tagged peptide (NBD‐FF‐pTyr) over 5 days. Representative images acquired daily are shown in Figure 7d. Green fluorescence from the NBD moiety indicated peptide self‐assembly within spheroids, with signal intensity gradually shifting toward the core over time.

The images demonstrate the time‐dependent distribution of the peptide, highlighting its ability to penetrate inner spheroid regions. Initially, fluorescence was localized near the surface. As time progressed, the signal intensified and became more uniformly distributed, suggesting peptide penetration into the spheroid core. These observations confirm the diffusion and internalization capability of the peptide, a critical feature for therapeutic efficacy in 3D tumor models, further supporting prior findings that NBD‐tagged peptides depend on cellular uptake, enzymatic activation, and redistribution. As shown in Figure 7f, relative fluorescence intensity increased from day 1 to day 3, reflecting progressive peptide accumulation. On subsequent days, however, a slight decrease was observed, possibly due to peptide redistribution within the spheroid, clearance or partial degradation. These quantitative results complement the qualitative imaging, confirming that peptide internalization occurs primarily during the early days of treatment.

### 3.7 Gene expression analysis

**FIGURE 8:**
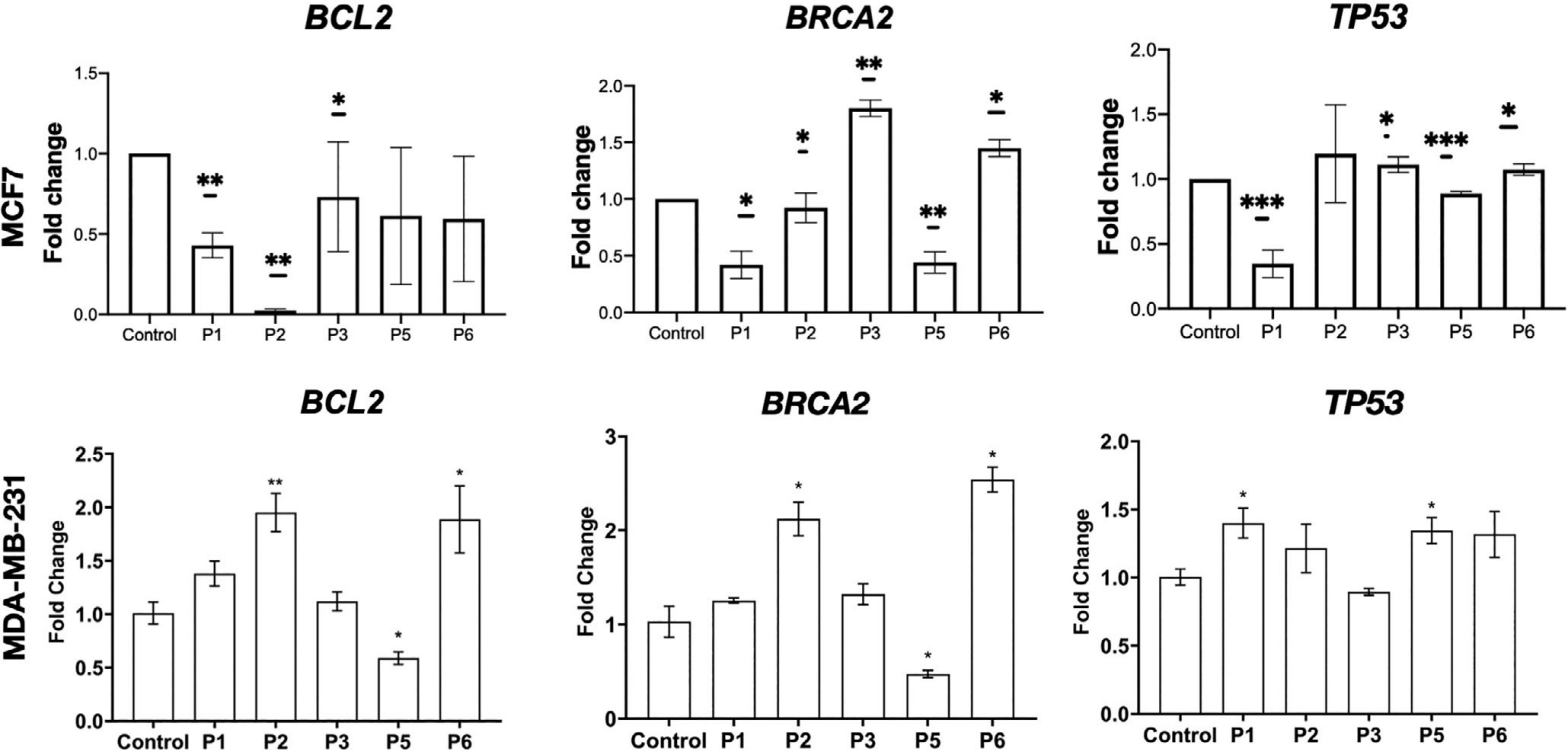
Gene expression of *BCL2*, *BRCA2*, *TP53* in MCF‐7 and MDA‐MB‐231 cells following treatment with five EISAPs (P1–P6) in static cultures. Fold changes were quantified by RT‐qPCR and normalized to GAPDH, with expression levels plotted relative to untreated controls.

Gene expression was examined in treated cells cultured under static conditions to determine peptide‐and cell line‐specific transcriptional response to stress and DNA damage. The RT‐qPCR data demonstrate that self‐assembling peptide treatments elicit distinct gene‐specific and cell‐line‐dependent transcriptional responses in breast cancer cells (Figure 8). In MCF‐7 cells, treatment with peptide P1 (Fmoc‐FF‐pTyr) markedly suppresses the ex‐pression of *BCL2*, *BRCA2*, and *TP53*, indicating a broad inhibitory effect on pathways regulating cell survival, genomic maintenance, and stress response. The downregulation of *BCL2* suggests reduced anti‐apoptotic signaling, making the cells more susceptible to pro‐grammed cell death. Simultaneously, decreased *BRCA2* expression points to an interruption of DNA‐repair capacity, which may impair homologous recombination and increase genomic vulnerability. *TP53* activity is primarily governed by post‐translational stabilization and protein‐level interactions (e.g., via the MDM2 axis). MCF‐7 cells harbor wild‐type tumor suppressive *TP53*, whereas MDA‐MB‐231 cells express a mutant form (R280K). In MCF‐7 cells (wild‐type *TP53*), the significant downregulation of *BCL2*, *BRCA2*, and *TP53* following treatment with P1 and P5 suggests a systemic collapse of the survival and DNA‐repair axes, priming these cells for apoptotic entry. Conversely, in the triple‐negative MDA‐MB‐231 line, which expresses a ‘gain‐of‐function’ mutant p53 (R280K), P1 treatment induced a compensatory upregulation of all three genes, signaling potential treatment resistance.

In MDA‐MB‐231, P5 demonstrated a superior therapeutic profile by successfully suppressing *BCL2* and *BRCA2* even in the MDA‐MB‐231 background. While TP53 mRNA levels remained elevated in these cells, the simultaneous knockdown of survival (*BCL2*) and repair (*BRCA2*) markers indicates that P5 may bypass the oncogenic protection typically afforded by mutant p53. P5 (Nap‐FF‐pTyr) therefore appears to be a promising therapeutic candidate capable of reducing key pro‐survival mechanisms, consistent with viability studies and our previous findings showing that P5 decreases mitochondrial activity.^7^

In contrast, P2 and P6 markedly elevate *BCL2*, suggesting increased survival signaling in this TNBC cell‐line. The upregulation of these genes indicates that the cells are undergoing stress responses while concurrently activating pathways involved in DNA repair and treatment resistance.

This behavior is typical of MDA‐MB‐231 cells, which are known for their resistance to DNA‐damaging anticancer agents. These increases, together with changes in *BRCA2* and *TP53*, suggest that the cells may activate compensatory resistance pathways to counteract peptide‐induced stress and maintain DNA integrity. P1 and P5 show to be promising therapeutics in reducing DNA repair pathways and overcoming resistance in cells.

## 4 CONCLUSIONS

This work underscores the value of 3D spheroid models on a pillar–plate platform as physiologically relevant, high‐throughput systems for evaluating cancer therapies under both static and dynamic conditions. Optimization of hydrogel composition was critical for spheroid stability and reproducibility, with alginate–gelatin achieving high transfer rates, a non‐invasive phenotype, and consistent performance.

Matrigel‐embedded spheroids modeled invasive behavior consistent with metastatic potential, while the Matrigel–alginate formulation delayed invasion and improved stability. Co‐treatment with DXR and the enzyme‐induced self‐assembling peptide Fmoc‐FF‐pTyr significantly reduced spheroid viability and invasion, particularly in Matrigel–alginate matrices, suggesting improved penetration and formation of finer nanostructures in the presence of alginate. This points to alginate as a supportive matrix for controlled drug delivery. Moreover, dynamic culture produced stronger synergistic effects of peptide–DXR co‐treatment with increased concentration and exposure time, resulting in a pronounced viability reduction observed at 48 h.

Furthermore, RT‐qPCR revealed subtype‐dependent transcriptional responses, indicating that peptide sensitivity is influenced by the breast cancer cell line. MCF‐7 spheroids showed downregulating effects on survival and repair genes after exposure to treatment. MDA‐MB‐231 spheroids, on the other hand, displayed a compensatory maintenance of survival pathways, highlighting the potential resistance mechanisms of TNBC.

Altogether, these results highlight the importance of employing EISAPs as targeted adjuvants to conventional chemotherapy, capable of enhancing efficacy and overcoming drug resistance in complex TMEs. Future studies should investigate the mechanisms underlying EISAP‐mediated cytotoxicity and assess their performance in heterogeneous spheroids incorporating stromal and immune cells, advancing EISAP‐based therapies toward preclinical evaluation.

## AUTHOR CONTRIBUTIONS

**Andrea Escobar Martinez:** Experimental design; Investigation; Data curation; Formal analysis; Literature search; Writing—original draft; Writing—review and editing. **Pranav Joshi:** Experimental design for spheroid formation; Methodology; Investigation. **Emily Carney:** Experimental design; Data interpretation. **Faye Fouladgar:** Experimental design; Data interpretation. **Robert Powell:** Experimental design. **Manav Vanga:** Experimental design; Methodology. **Victoria Gnenema:** Spheroid formation; Investigation. **Sarah Hripko:** Experimental design. **Moo‐yeal Lee:** Conceptualization; Supervision. **Neda Habibi:** Conceptualization; Supervision; Funding acquisition; Writing—review and editing. All authors contributed to manuscript review and approved the final version of the manuscript.

## FUNDING INFORMATION

This work was supported by the National Institute of General Medical Science (NIGMS) of the National Institutes of Health under award number R16GM150848.

## CONFLICT OF INTEREST STATEMENT

The authors declare no conflicts of interest.

## CONSENT

All authors agreed with the content of this manuscript and approved the submitted version.

## Data Availability

The data that support the findings of this study are available from the corresponding author upon reasonable request.
